# Three-Dimensional Printed Porous Titanium Screw with Bioactive Surface Modification for Bone–Tendon Healing: A Rabbit Animal Model

**DOI:** 10.3390/ijms21103628

**Published:** 2020-05-21

**Authors:** Yu-Min Huang, Chih-Chieh Huang, Pei-I Tsai, Kuo-Yi Yang, Shin-I Huang, Hsin-Hsin Shen, Hong-Jen Lai, Shu-Wei Huang, San-Yuan Chen, Feng-Huei Lin, Chih-Yu Chen

**Affiliations:** 1 Department of Biomedical Engineering, National Taiwan University, Taipei 106, Taiwan; yellowcorn0326@yahoo.com.hk (Y.-M.H.); judyya1022@gmail.com (S.-W.H.); double@ntu.edu.tw (F.-H.L.); 2 Department of Orthopedics, Shuang Ho Hospital, Taipei Medical University, Taipei 100, Taiwan; 3 Department of Orthopedics, School of Medicine, College of Medicine, Taipei Medical University, Taipei 100, Taiwan; 4 Department of Materials Science and Engineering, National Chiao-Tung University, Hsinchu 300, Taiwan; Sigher@itri.org.tw (C.-C.H.); sanyuanchen@mail.nctu.edu.tw (S.-Y.C.); 5 Biomedical Technology and Device Research Laboratories, Industrial Technology Research Institute, Hsinchu 310, Taiwan; peiyi@itri.org.tw (P.-IT.); yangkuoyi@itri.org.tw (K.-Y.Y.); SophiaShinI@itri.org.tw (S.-IH.); shenhsin@itri.org.tw (H.-H.S.); 6 Material and Chemical Research Laboratories, Industrial Technology Research Institute, Hsinchu 310, Taiwan; hjlai@itri.org.tw; 7 Institute of Biomedical Engineering and Nanomedicine, National Health Research Institutes, Miaoli County 360, Taiwan

**Keywords:** bioactive ceramic coating, interference screw, additive manufacturing, titanium-alloy implant

## Abstract

The interference screw fixation method is used to secure a graft in the tibial tunnel during anterior cruciate ligament reconstruction surgery. However, several complications have been reported, such as biodegradable screw breakage, inflammatory or foreign body reaction, tunnel enlargement, and delayed graft healing. Using additive manufacturing (AM) technology, we developed a titanium alloy (Ti_6_Al_4_V) interference screw with chemically calcium phosphate surface modification technology to improve bone integration in the tibial tunnel. After chemical and heat treatment, the titanium screw formed a dense apatite layer on the metal surface in simulated body fluid. Twenty-seven New Zealand white rabbits were randomly divided into control and additive manufactured (AMD) screw groups. The long digital extensor tendon was detached and translated into a tibial plateau tunnel (diameter: 2.0 mm) and transfixed with an interference screw while the paw was in dorsiflexion. Biomechanical analyses, histological analyses, and an imaging study were performed at 1, 3, and 6 months. The biomechanical test showed that the ultimate pull-out load failure was significantly higher in the AMD screw group in all tested periods. Micro-computed tomography analyses revealed early woven bone formation in the AMD screw group at 1 and 3 months. In conclusion, AMD screws with bioactive surface modification improved bone ingrowth and enhanced biomechanical performance in a rabbit model.

## 1. Introduction

Anterior cruciate ligament (ACL) tears are a common sports injury, and ACL reconstruction (ACLR) is the most performed procedure to restore knee stability to enable a return to sports [1]. The success of ACLR depends on graft healing and the type of fixation. Tibial fixation is commonly considered weaker than femoral fixation because the bone mineral density of the proximal tibia is lower than that of the distal femur [2,3]. Furthermore, the force vector of ACL grafts is parallel to the tibial tunnel, which places maximal force on the tibial graft fixation [2]. There are two major categories of interference screws used for ACLR, namely permanent metal screws and bioabsorbable screws. Bioabsorbable screw materials include polyglycolic acid, poly-L-lactic acid, and poly-D,L-lactic acid. Clinical outcomes have been equivalent after the use of bioabsorbable screws and metal interference screws for tibial graft fixation [4]; however, higher rates of effusion, screw breakage, and incomplete tunnel healing have been reported for bioabsorbable screws compared with titanium alloy (Ti) interference screws [5]. The rate of clinical sequelae from bioabsorbable tibial interference screws was high, with symptoms in approximately 1 of 10 ACLRs in adolescents [6]. Therefore, in this rabbit study, we selected Depuy Synthes Ti cortical screws as the control groups for tibial intratunnel healing and compared them with experimental screws of the same diameter and similar morphology. We evaluated the effects of Ti interference screws on tibial bone–tendon graft incorporation.

Titanium is a widely used metallic biomaterial with excellent fatigue and corrosion resistance [7]. To enhance bone integration, the modification of surface properties is thought to be able to improve the interaction and healing of tissue materials. White et al. [8] reported that porous materials can induce osteointegration. Clemow et al. [9] demonstrated that different Ti pore sizes can alter the shear force and bone ingrowth. Porous Ti samples stimulated faster osteoblast cell differentiation and ingrowth because of cell morphology changes within the Ti pores [10]. To augment further cell adhesion and tissue ingrowth, research has been devoted to bioactive ceramics on Ti implants [11]. Bioactive ceramics have a surface similar to the structure of bone and the ability to enhance bone tissue formation [12]. Plasma-sprayed hydroxyapatite (HA) coated on Ti has been commonly used; however, the heating process during coating results in undesirable HA decomposition and altered biocompatibility [13]. Moreover, Yang et al. reported that the different thermal expansion coefficients of HA and Ti_6_Al_4_V result in high tensile stress and poor adhesion [14]. Furthermore, differences in HA thickness may cause fractures inside the HA coating layer or at the HA–Ti interface [15]. Coating Ti implants with bioactive calcium phosphate (CaP) ceramic provides an osteoconductive surface that stimulates bone ingrowth and minimizes prosthesis loosening [11,16]. Aniket et al. reported that bioactive ceramic coatings can induce osteoblast-like cell accumulation and decrease inflammatory bone cell responses [17]. Tadashi et al. reported a novel bioactive titanate layer using chemical and heat treatments [18]. When Ti alloy was soaked in CaCl_2_ solution after NaOH treatment, the sodium hydrogen titanate formed by NaOH was transformed into calcium hydrogen titanate. After heat treatment and soaking in simulated body fluid (SBF), the apatite formed on the surface of Ti because positively charged calcium ions and negatively charged phosphate ions formed CaP. After a subsequent water treatment, the titanate formed bone-like apatite on the surface of the titanate in SBF.

Implant designs can be improved through additive manufacturing (AM) technology, which offers engineers new design freedom [19]. AM technology, otherwise known as three-dimensional (3D) printing, is a process through which a 3D object is created by successively layering materials, such as liquids, biodegradable polymers, or metals. AM technology has been used for a controlled drug delivery system [20], regenerative medicine [21], and joint replacement design [22]. AM technologies are superior to conventional fabrication techniques for producing porous structures for bone ingrowth [23,24,25]. Furthermore, AM technology provides greater customizability, speed, and accuracy through computer-aided design. In our previous study, we illustrated how a porous Ti interference screw produced using AM technology could improve tibial graft fixation and enhance the biomechanical performance of the bone–tendon–screw construct [26]. We proposed that by using surface modification technology, we could develop a novel interference screw with appropriate structural porosity and surface reactivity, which would improve graft fixation and enhance biomechanical performance.

## 2. Results

### 2.1. Additive Manufactured Interference Screw with Bioactive Surface Modification

**Figure 1:**
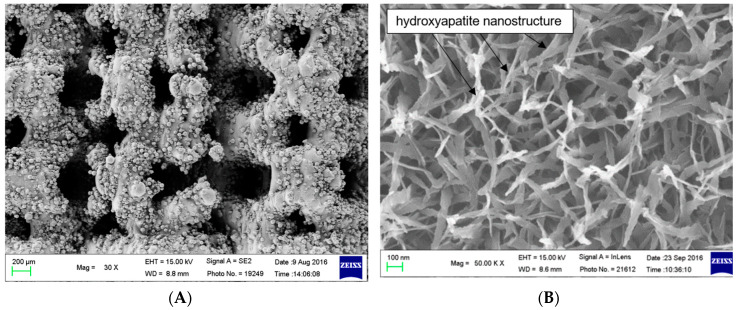
(**A**) Outer surface of the additive manufactured screw, low magnification. Scanning electron microscopy images of the surface of the additive manufactured screw, (**B**) Bioactive surface coating, high magnification. The SEM images reveal a diffuse feather-like apatite nanostructure coating on the Ti metal, which had been soaked in SBF for 3 days after NaOH and heat treatments.

In Figure 1, scanning electron microscopy (SEM) images clearly show a feather-like apatite nanostructure coated onto the surface of the additive manufactured (AMD) screw after bioactive surface modification. The apatite nanostructure had a diameter of approximately 50–100 nm, the thickness of the coating layer was approximately 1 µm, and the average pore size on the AMD screw was from 200 to 400 µm.

**Figure 2:**
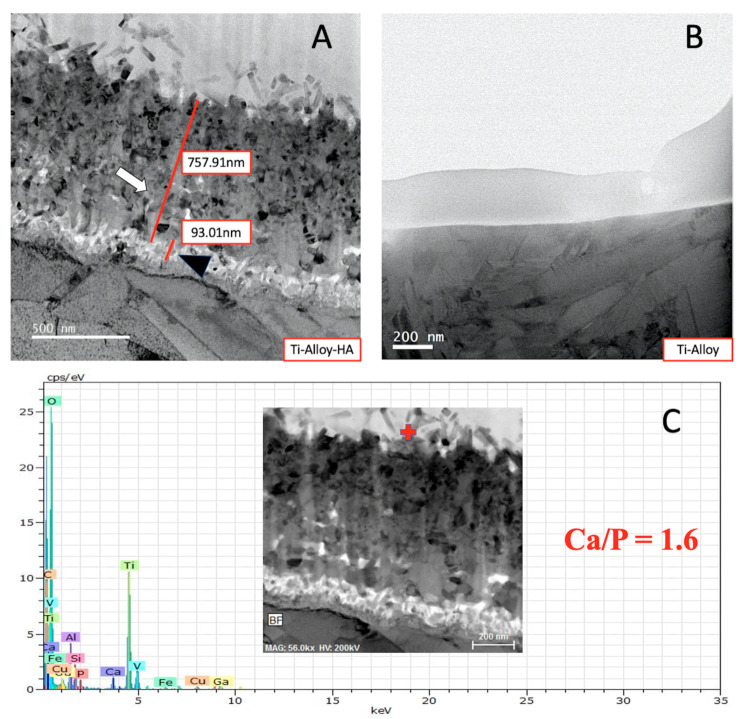
TEM photograhs and EDX examination. (**A**) Ti-alloy with surface modification, formation of apatite can be seen on the Ti-alloy surface (white arrow), black arrowhead denotes anatase and rutile on the Ti-alloy surface. (**B**) Ti-alloy only. (**C**) EDX on the apatite of the Ti-alloy with surface modification. Red cross marks the area of electron diffraction and EDX analysis.

In Figure 2, transmission electron micrograph (TEM) images illustrate the apatite formation on the AMD screw after surface modification. Bone-like apatite is formed on the surface of Titaum metal in SBF. The apatite formation on the surface layer is needle-like form, and has a composition with Ca/P ration = 1.6 from the transmission electron micrograph and energy dispersive X-ray spectroscopy (EDX) (Figure 2C).

### 2.2. Biomechanical Analysis

**Figure 3:**
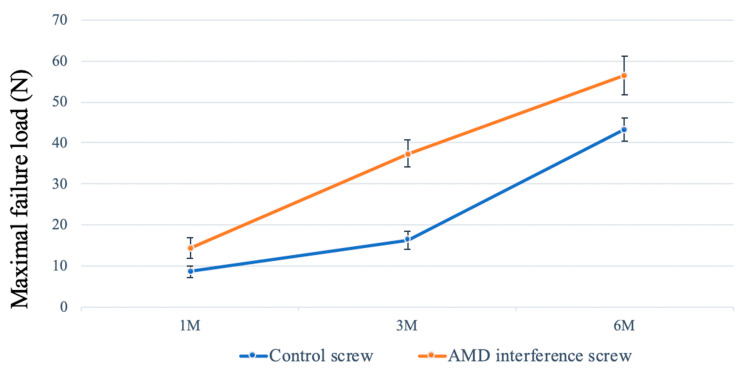
Biomechanical assessment. The mean maximal failure load in the AMD screw group was significantly higher than that in the control group at 1, 3, and 6 months (*p* < 0.05). The error bar denotes the standard deviation.

In the biomechanical test, the ultimate load-to-failure in the AMD screw was consistently significantly higher than that in the control group at 1, 3, and 6 months (Figure 3). All specimens failed at the bone–tendon–screw junction in the tibial tunnel. The mean maximal load at failure was 43.3 ± 2.7 N in the control group compared with 56.6 ± 4.7 N in the AMD group in 6-month specimens (*p* = 0.0001). In both groups, the maximal failure load increased progressively during the study period. The increase in strength was correlated with more bone ingrowth and improved bone–tendon healing.

### 2.3. Micro-Computed Tomography Analysis

**Figure 4:**
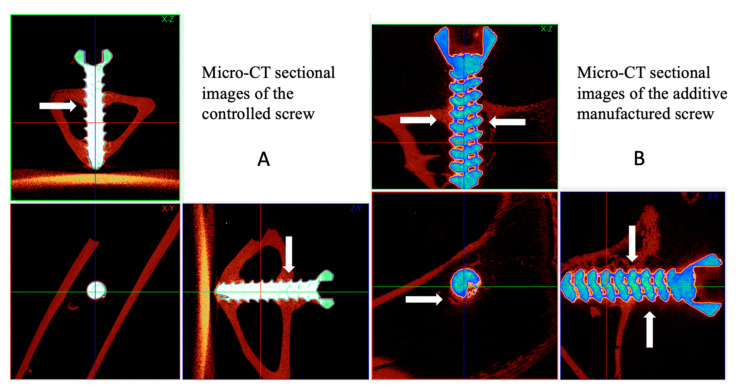
Micro-computed tomography (Micro-CT) examination. Comparison of micro-CT sectional images (**A**,**B**) between the interference screws. Micro-CT revealed woven bone formation between the AMD screw and bone tunnel in all sectional images. The AMD screw was fixed in the bone tunnel with massive bone ingrowth from the cancellous bone of the proximal tibia. Line arrows mark the woven bone formation.

New bone formation between the implant and bone tissue was evaluated using micro-computed tomography (micro-CT), and the bone volume/tissue volume (BV/TV, %) and bone surface area/total volume (BS/TV, 1/mm) were quantified. The bone volume fraction and bone surface density were measured 100–1000 µm exterior to the implant, which represented the bone volume rate and bone surface rate for woven bone formation. Figure 4 illustrates dense new bone formation around the controlled and AMD screw in the tibial bone tunnel. The woven bone formed diffusely around the AMD screw with bone healed from the cancellous bone in the bone tunnel.

**Figure 5:**
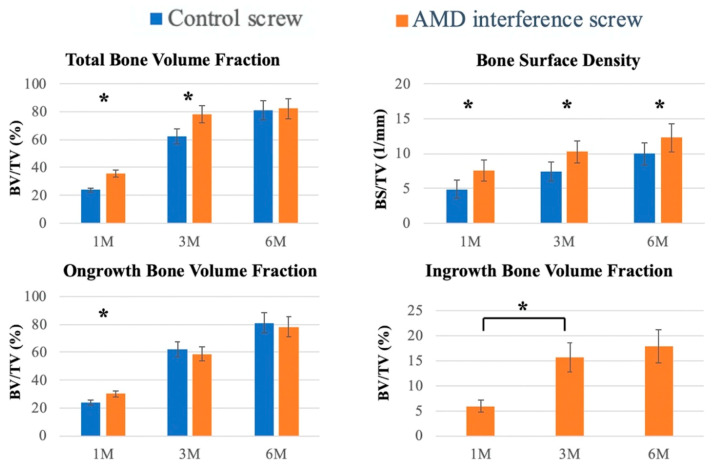
Quantitative evaluation of bone volume between bone and screw. interfaces. Micro-CT was used to evaluate the bone ingrowth after screw implantation. Tissue volume (TV: mm^3^), bone volume (BV: mm^3^), and bone surface (BS: mm^2^) were retrieved from 100 to 1000 µm above the implant. Bone volume fraction (BV/TV) and bone surface density (BS/TV) represent the bone volume rate and bone tissue surface rate, respectively. Ongrowth bone volume denotes the bone outside the control screw, and ingrowth bone volume denotes bone mass inside the surface of the implant; the error bar represents the standard deviation, and * denotes statistical significance between the two groups.

In the quantitative evaluation of bone volume, we used a nonporous control screw as a template. Outer bone growth was defined as that exterior to the nonporous implant, and inner bone growth was defined as the bone inferior to the surface of the implant. The total bone volume was calculated using the accumulation of outer and inner bone growth. The total bone volume fraction (BV/TV) was significantly higher in the AMD group at the 1- and 3-month follow-ups (Figure 5; *p* < 0.001). The total bone surface density was significantly higher in 1-, 3-, and 6-month specimens for the AMD interference screw group. The ongrowth bone volume fraction was significantly higher in the AMD screw group at the 1-month follow-up. Because the control group did not have a porous structure, only the AMD screw group had bone ingrowth inferior to the surface of the implant. Subsequently, we evaluated the differences in the percent of ingrowth bone volume between different time points, and it was statistically significant between the 1- and 3-month follow-ups. No differences existed in outcomes for ingrowth bone surface density.

### 2.4. Histology Analysis

**Figure 6:**
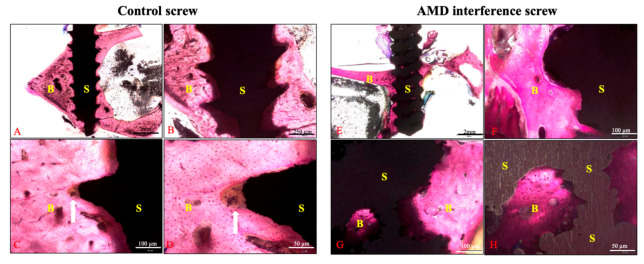
Histological examination of the bone–screw interface between groups. The specimens were stained with Sanderson’s Rapid Bone Stain and then counterstained with acid fuchsin. Figures (**A**–**D**) represented the controlled screw from scale of 12.5×, 40×, 100×, and 200×, respectively. The surface of the controlled screw was sharp and healed with fibrous tissue at the tip of screw thread. The AMD screw group (**E**–**H**) exhibited a rougher surface and superior bone–screw integration on the screw surface and inside the screw body. Line arrows indicate fibrous tissue healing at the tip of the control screw thread; B: bone, S: screw.

In the histological examination, superior bone–screw integration was observed in the AMD screw group (Figure 6). The bioactive ceramic coating on the AMD screw makes the screw surface rough, which readily promotes bone ingrowth. In the control screw group, healing was identified at the bone–screw interface, where fibrous tissue covered the tip of the screw thread (Figure 6C,D). Moreover, bone ingrowth inside the AMD screw was identified as being because of the porous structure inside the screw (Figure 6G,H).

**Figure 7:**
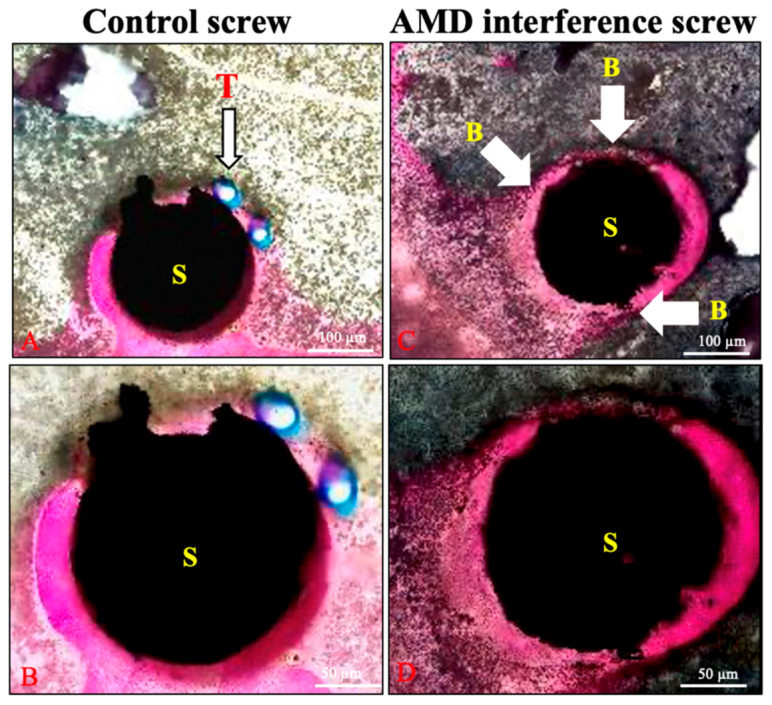
Histological examination of the 6-month specimens. More woven bone formation (wide arrows) was identified in the bone–screw construct in the AMD screw group. The tendon graft (line arrow) is shown between the screw and bone in the control group (**A**,**B**). The bone and screw were in close contact in the AMD screw group (**C**,**D**); B: bone, T: tendon, and S: screw.

In the AMD screw group, the bone–screw interface was healed with more woven bone (Figure 7). The gap between the bone–screw construct was wider in the control group comparing with the AMD screw group. These findings were compatible with the micro-CT findings.

## 3. Discussion

In this study, we designed an innovative AMD Ti screw with a ceramic surface modification for an animal study. For the biomechanical test, the AMD screw had a much stronger ultimate failure strength than the control, which indicated superior bone ingrowth in the bone–tendon–screw construct. A rough surface and porous structure have been reported to improve bone healing and reduce micromotion after screw implantation [19,27]. Such results are compatible with our study, where the total bone volume fraction was significantly higher at the 1- and 3-month follow-ups, which is crucial for graft healing after ACLR surgery. Because of fibrous tissue between the tendon–bone interface, a weak connection exists between tendon grafts and bones in ACLR, especially in the first 6 weeks [28]. Moreover, the bone surface density was significantly higher in our AMD screw group at all follow-ups, presenting more woven bone formation on the surface of the implants. These results not only reflect the porous structure on the AMD screw but also the surface modification that occurred with CaP apatite. In the SEM images, the feather-like apatite evident on the surface improved the bone tissue integration. This was also proved by the histological images, where the bone was observed to have healed to the screw more prominently instead of fibrous tissue forming. Our findings indicated that the AMD screw with ceramic bioactive surface modification could improve early woven bone formation and implant stability with a stronger ultimate pull-out load.

Surface modification has been demonstrated to be an effective strategy to accelerate bone healing at early implantation times. Coating with a layer of HA is one of the most commonly used methods because of its excellent biocompatible and osteoconductive properties [29]. Coating orthopedic Ti_6_Al_4_V with HA produces an osteoconductive surface that promotes early bone integration and minimizes the prosthesis loosening rate [30]. The capacity of HA to immobilize growth factors and proteins through noncovalent interactions improves the healing process [31,32]. However, delamination and fragmentation of HA has been reported [33], either caused by structural defects between the coating and implant or structural nonhomogeneity in terms of thickness and crystallinity [14]. The difference in the thermal expansion coefficient between HA and the implant induces interface stress and further poor adhesion [14]. Moreover, plasma spraying of HA does not produce a completely crystalline layer, which can cause the amorphous component to disappear from the HA coating, leading to its instability [34]. Wet chemical techniques can be used to form a thin film with strong bonding of the CaP coating and reduce the risk of film delamination and inflammatory reactions [35]. The surface-modified material is immersed in a CaP solution, such as SBF with pH and ion concentrations equal to human blood plasma [36]. Wet chemical techniques allow the immobilization of biofunctional substances within the CaP layer, such as bone morphogenetic protein [37], proteins [38], vitamins [39], and antibacterial agents [40]. Nguyen et al. demonstrated that sol–gel-formed CaP coatings improve bone ingrowth and increase bone-to-implant contact [35]. Nishiguchi et al. demonstrated that alkali- and heat-treated Ti result in strong bone bonding and a high ongrowth rate [41]. In another study, the osteoconduction of CaP coating was initiated with decreased local pH and partial dissolution of the coating for calcium and phosphate release [42]. The ions’ reprecipitation and incorporation of proteins stimulated chemotaxis for bone conduction. In our study, we demonstrated enhanced and early bone ingrowth in the AMD screw with a bioactive ceramic surface modification, a finding that demonstrated a synergistic relationship between the CaP modified surface and bone tissue.

AM has great potential for personalized medicine in bone scaffolds and orthopedic implants [19]. AM techniques have been employed for orthopedic applications, such as the reconstruction of hips [23], knees [43], and clavicles [44]. The advantages of AM are that it creates porous and rough surfaces, which induce bone ingrowth [24]. Higher friction resistance may cause lower micromotions of the bone–implant construct for enhanced tissue integration [45]. Moreover, different pore sizes were shown to alter the progression of osteogenesis [46]. Significant differences in bone volume fraction and bone surface rate suggested superior bone ingrowth especially on the surface of the AMD screw. Bone growth clearly increased the stability of the implant and enhanced mechanical load failure. In our study, the AMD screw with surface modification was able to improve healing in the bone–tendon–screw construct. AM offers new possibilities for improving long-term metal implant fixation. Richard et al. showed that using additive manufactured method could achieve maximal pull-out force with optimal design [47]. Bone is a 3D inhomogeneous structure which can be classified as compact bone (cortical bone) and trabecular bone (cancellous bone) with different porosity [48]. Moreover, the Young’s modulus of human bone is much lower than the solid metals. AM technique can provide new design with maximal porous design and adequate resistance force, which is very difficult to accomplish through traditional method [19].

Although our results are encouraging, this study still had some limitations. First, the AMD interference screw had a similar morphology to the control screw. However, the nonporous structure and smooth surface of the commercial Ti screw were different from the AMD screw. Relevant studies have reported differences in pore size and surface roughness integration [49]. However, the ideal pore size of AMD screws has not been evaluated. The minimum pore size is considered to be approximately 100 µm because of cell size, migration, and transport. However, larger pore sizes are considered to enhance capillary formation and osteogenesis [46]. Second, this study used an extra-articular animal model, which differed from ACLR. The axis of the tibial tunnel was different, and the graft tunnel was not connected to the knee joint. Therefore, a positive effect in this model could not be confirmed for ACLR. Further animal studies using AMD screws with bioactive coating should be designed.

## 4. Materials and Methods

### 4.1. Study Design

In accordance with national animal welfare legislation and the National Institute of Health guidelines for the use of laboratory animals, this animal study was pre-approved by the Ethics Committee of the Industrial Technology Research Institute, Biomedical Technology and Device Research Laboratories, Taiwan (Approval No. MI-20190602, 03 June 2019). Twenty-seven New Zealand white rabbits (Master Laboratory Co., Ltd., Taipei City, Taiwan) with a mean body weight of 3.2 ± 0.4 kg and age of 6 months were selected. Using computer-generated randomization, a traditionally made Ti screw was implanted in one of the stifle joints (the control group) and an AMD porous screw with bioceramic coating was implanted in the other stifle joint of the same rabbit (the experimental group). All rabbits were divided into three groups based on implantation periods of 1, 3, and 6 months (nine in each group). In each group, three rabbits were used for the histological analysis, and the other six rabbits were used for micro-CT and biomechanical studies. For each animal, micro-CT analyses were performed immediately at the end of each experiment, and then the specimens were fresh-frozen for further biomechanical study.

### 4.2. Production of the Innovative Interference Screw

**Figure 8:**
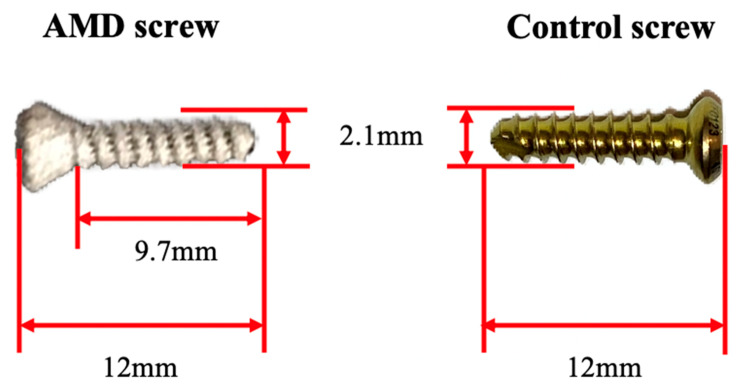
Illustration of a porous AMD screw. The control interference screw was made with Ti and had a screw body length of 12 mm, core diameter of 1.8 mm, thread diameter of 2.1 mm, head diameter of 2.4 mm, and pitch of 1.0 mm (Depuy Synthes, Johnson and Johnson, New Brunswick, NJ, USA). The AMD screw with a similar morphology was produced using an EOSINT M 280 laser sintering system (EOS GmbH, Munich, Germany). The AMD interference screws received NaOH and heat treatments for bioactive ceramic surface modification.

This study’s controlled Ti screws were obtained from an AO cortical screw (DePuy Synthes, Johnson and Johnson, New Brunswick, NJ, USA) with a screw body length of 12 mm, core diameter of 1.8 mm, thread diameter of 2.1 mm, head diameter of 2.4 mm, and pitch of 1.0 mm, as shown in Figure 8.

The AMD Ti-6Al-4V screws were fabricated using selective laser melting, and a porous structure with a porosity of approximately 15.4% was formed during the AM process with the EOSINT M 280 system. The AMD screw was a template and evolved from the control screw with identical mechanical parameters of length, core diameter, thread diameter, head diameter, and pitch. Subsequently, the outer surface of the AMD screw was modified through a bioactive ceramic coating. Bioactive surface modification with porous Ti was performed using a chemical and heat treatment method. The main processing procedure was as follows. The sample was (1) ultrasonically cleaned; (2) soaked in 1 M NaOH solution at 60 °C for 24 h; (3) soaked in 100 mM CaCl_2_ solution at 40 °C for 24 h; (4) cleaned with distilled water and dried; (5) heated to 600–800 °C for 1 h at a heating rate of 5 °C /min; (6) after cooling, it was soaked in alkaline pH 7.4 SBF containing NaCl, NaHCO_3_, KCl, K_2_HPO_4_, MgCl_2_, CaCl_2_, and Na_2_SO_4_ for 3 days.

After chemical surface modification, the AMD screws formed a dense and uniform bone-like apatite layer (approx. thickness = 1 μm) on the metal surface in SBF solution. This apatite coating was expected to enable the use of bioactive Ti-6Al-4V implants as artificial bones to enhance bone growth. Figure 7 presents a comparison of the control and AMD screws with bioactive ceramic coating.

### 4.3. Surgical Procedures

All rabbits received a preoperative dose of intramuscular cefazolin sodium (0.1 mg/kg) as a prophylactic antibiotic. A Zoletil–Rompun mixture (Zoletil 15 mg/kg [Virbac Taiwan, Taiwan] + Rompun 0.05 mL/kg [Bayer Taiwan, Taiwan]) was intramuscularly injected to induce general anesthesia. For analgesia, the rabbits were administered meloxicam (0.15 mg/kg peroral; Metacam, Boehringer Ingelheim Taiwan, Taiwan) 1 day preoperatively, immediately preoperatively, and 2 days following surgery.

We used an animal model similar to that developed by Yamakado et al. [50] with some modifications. First, we made a lateral parapatellar approach on the bilateral knee to approach the lateral femoral condyle. The tendon of the extensor digitorum longus (EDL) was detached from its origin. A drill hole (diameter: 2.0 mm) was created using an electrical drill at the proximal tibial metaphysis perpendicular to the long axis of the tibia. The EDL tendon was passed through the bone tunnel and transfixed with the interference screw from the medial to lateral side while the paw was supported in dorsiflexion. We repaired the joint capsule, muscles, and fascia with 4-0 Vicryl sutures. After surgery, the rabbits were allowed to freely exercise in the same cage without restrictions. All animals were ambulant without any protection or immobilization. Euthanasia was performed through injecting an intravenous overdose of pentobarbital. Stifle joints were stored at −20 °C for further analyses.

### 4.4. Microscopic Observation

Microscopic observation was carried out using a scanning electron microscope (SEM; TM 1000, Hitachi) and the morphology of bioactive ceramic coating was observed. SEM observation was with an operating voltage of 15 kV and the vacuum level of the observation chamber was 10^-5^ to Pa. Transmission electron micrograph (TEM) and energy dispersive X-ray analysis (EDX) was used to evaluate the chemical component of bioactive apatite. TEM images were obtained using a JEOL transmission electron micrograph (JEM-2100F, JEOL, MA, USA) at 200 kV. Energy Dispersive X-ray analysis was used to identify the chemical element of bioactive apatite. EDX measurement were performed on a JSX-1000S Fluorescence Spectrometer X-ray analyzer (JEOL, MA, USA). The EDX spectrum was obtained at an acceleration of 20 kV and collected for 19 seconds.

### 4.5. Biomechanical Analysis

Six rabbits from each group were selected for a biomechanical test. We carefully dissected the EDL tendon from the distal end of insertion and removed all other redundant tissue. We used a material testing machine (ElectroForce 3510-AT; Bose Corporation, Framingham, MA, United States) for biomechanical testing. The graft was secured on the clamps at a distance of 3 cm from the lateral tibial aperture, allowing tensile loading along the long axis of the tibial bone tunnel at a strain rate of 0.5 mm/min until failure occurred. The ultimate pull-out load and failure mode were recorded and analyzed.

### 4.6. Micro-Computed Tomography Analysis

Six rabbits from each group were sacrificed at 1, 3, and 6 months postoperatively. Micro-CT was used to evaluate the new bone formation in the bone–screw interface. The specimens were prepared for micro-CT evaluation (Skyscan 1176 µCT System; Bruker, Kontich, Belgium). High-voltage scanning (voltage: 90 kVp, current: 278 µA, at a 25 W output with a 360° scan) and an 18-µm sample size were used for this experiment. Cross-sectional images were reconstructed, and the region of interest was further selected. We performed the analyses with 1.5 mm specimen for 100 slice images. The tissue volume (TV, mm^3^), bone volume (BV, mm^3^), bone volume fraction (BV/TV, %), bone surface (BS, mm^2^), and bone surface rate (BS/TV, 1/mm) were retrieved from 100 to 1000 µm above the metal implant. We used a nonporous implant as a template; outer bone growth was defined as that exterior to the nonporous implant surface, and inner bone growth was defined as the bone formation interior to the surface of the implant. The total bone growth indicated the sum of the outer and inner bone growth.

### 4.7. Histological Analysis

The last three rabbits were sacrificed for histological examination. The specimens were fixed with 10% formalin for several days and decalcified with graded series of alcohol. The proximal tibia was embedded in paraffin and sliced into approximately 150-µm slices using an IsoMet Low Speed Saw (Buehler, Lake Bluff, IL, United states) and ground to 60 µm with a grinding/polishing machine. The samples were sectioned perpendicular to the long axis of the tibia tunnel. Staining was performed using Sanderson’s Rapid Bone Stain (Dorn and Hart Microedge, Loxley, AL, USA) and then counterstained with acid fuchsin. Histological sections were examined using a light microscope (Nikon ECLIPSE, Melville, NY, USA).

### 4.8. Statistical Analysis

Before the animal study, a power analysis was performed to calculate the number of animals needed to prevent type II errors. In our previous study, micro-CT revealed the bone volume fraction (BV/TV) to be 39.0 ± 6.7 % for the experimental tibia [26]. Six specimens per group achieved a power of 0.80 with α = 0.05. All data were expressed as mean ± standard deviation. The obtained biomechanical test and CT data were compared using paired *t* tests. All statistical analyses were performed using the SPSS 18.0 software package (SPSS, Chicago, IL, USA). The results were considered statistically significant at *p* < 0.05.

## 5. Conclusions

In this study, a bioactive ceramic coating was produced on Ti metal using chemical and heat treatments. The AMD screw achieved a higher ultimate pull-out strength and a larger amount of woven bone formation than the control; furthermore, the innovative screw implantation in the rabbit model resulted in enhanced bone integration and stability in the bone–tendon–screw construct.
